# Primary Intraosseous Malignant Peripheral Nerve Sheath Tumor of the Humerus: Report of a Rare Case

**DOI:** 10.7759/cureus.33178

**Published:** 2022-12-31

**Authors:** Wendolin J Ortiz, Maria S Salazar, John J Eager, Saleha Sajid, Mario Cervantes

**Affiliations:** 1 Pathology, HCA (Hospital Corporation of America) Houston Healthcare West, Houston, USA; 2 General Surgery, Universidad Autónoma de Baja California, Mexicali, MEX; 3 Pathology, Universidad de Oriente, Ciudad Bolivar, VEN; 4 Orthopaedic Surgery, HCA (Hospital Corporation of America) Houston Healthcare West, Houston, USA; 5 Oncology, Genesis Medical Group, Houston, USA; 6 Pathology, HCA (Hospital Corporation of America) Houston Healthcare Pearland, Houston, USA

**Keywords:** sox10, sarcoma, proximal humerus, mpnst, malignant peripheral nerve sheath tumour

## Abstract

Malignant peripheral nerve sheath tumors (MPNSTs) usually arise in the soft tissues. Intraosseous MPNSTs are rare. They may arise *de novo *but are typically associated with neurofibromatosis type 1 (NF1) and radiation therapy. Our patient is a 58-year-old female patient that presented with right shoulder pain. An MRI showed a shoulder mass, and percutaneous bone biopsy demonstrated morphology suggestive of an MPNST; besides, on immunohistochemistry, SOX10 was positive, and H3K27me3 expression was entirely lost. The patient underwent total resection of the right proximal humerus and endoprosthetic shoulder reconstruction, followed by radiation therapy and chemotherapy. Only a few cases in the mandible, spine, maxilla, ulna, metacarpal, tarsal, and one in the humerus have been published. In this paper, we contribute with an additional case of primary intraosseous MPNST in the humerus and a brief literature review.

## Introduction

Primary intraosseous malignant peripheral nerve sheath tumors (MPNSTs) are rare, highly aggressive tumors arising from peripheral nerves, usually present as soft tissue masses with pain or loss of function. Patients complain of constant pain and are more likely to have motor and sensory deficits in the distribution of the parent nerve. They usually affect adults in the third to fifth decades of life and are frequently associated with neurofibromatosis (NF) type 1 and 2 or previous radiotherapy treatment. About 50% of MPNSTs occur *de novo. *The most common sites for MPNSTs are the peripheral nerves of the trunk, extremities, head and neck regions, and spine. Histologically, these tumors are highly cellular with frequent mitoses and exhibit anaplasia, necrosis, infiltrative growth pattern, pleomorphism, and high proliferative activity. Most MPNSTs are high-grade and stain for the S100 protein; weak staining is associated with a higher risk of distant metastasis. NF1-associated MPNSTs have a worse outcome compared to patients with sporadic MPNST. Treatment for peripheral nerve tumors is surgical resection [1-3].

## Case presentation

Our patient is a 58-year-old woman with a history of hypertension, hyperlipidemia, osteopenia, gastric bypass, and hysterectomy. The patient had been complaining of right shoulder pain for several months; she rated the pain as 3/10 in intensity, intermittent, aching, sharp, and throbbing in nature, aggravated by position. She had a magnetic resonance imaging (MRI) of the right shoulder performed one year prior, which showed a shoulder mass. The patient mentioned that she had only been using heating pads or acetaminophen for the pain.

A right proximal humeral mass was found on physical examination, and radiographs showed a malignant lesion with a somewhat nonspecific radiographic appearance. An MRI and computerized tomography (CT) scan were ordered. The MRI of the upper extremity without contrast showed a 3.9 x 2.6 x 7.7 cm neoplasm in the proximal right humeral meta diaphysis, disrupting the posterior cortex. A CT of the chest, abdomen, and pelvis with contrast showed a lytic lesion involving the proximal right humerus measuring up to 4.4 x 3.6 x 3.5 cm. 

**Figure 1:**
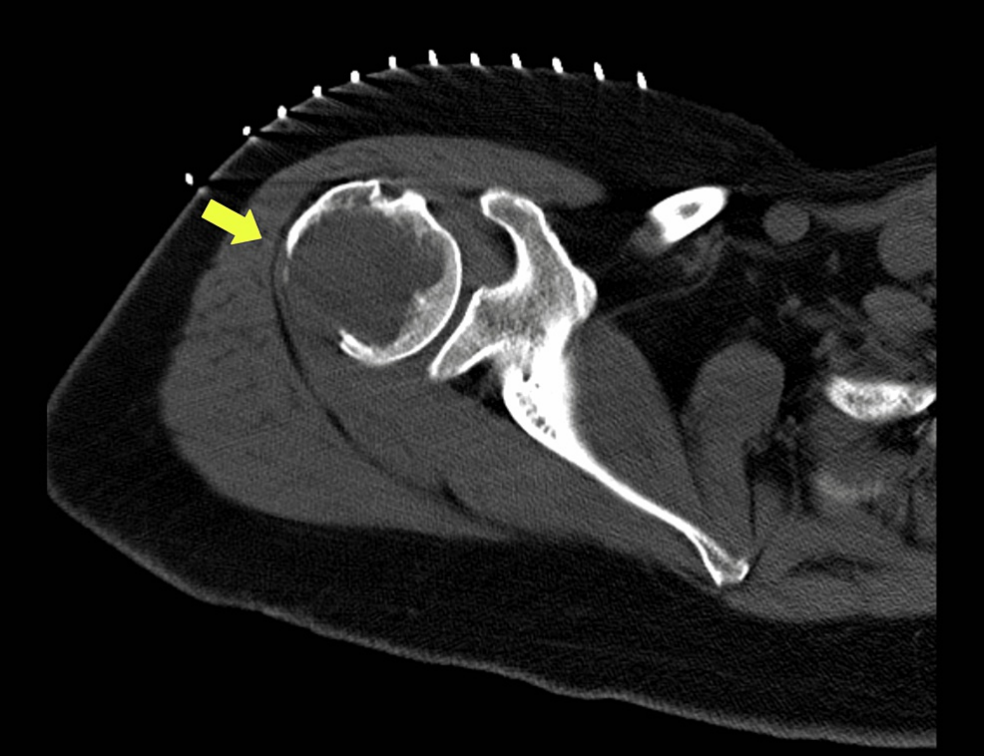
Percutaneous bone biopsy of lytic lesion involving the proximal right humerus

The next day, a percutaneous bone biopsy was performed to study the lesion further (Figure 1).

Per pathology, intersecting fascicles of monomorphic spindled cells with wavy or buckled nuclei associated with cells showing granular cell change were found. On immunohistochemistry (IHC), complete loss of H3K2me3 was seen; S100 protein, SOX10, Desmin, Myogenin, MyoD1, and CD34 were negative. A diagnosis of high-grade spindle cell sarcoma showing morphologic and IHC features of an MPNST was made. 

The patient was scheduled for total resection of the right proximal humerus and endoprosthetic shoulder reconstruction. The patient underwent a radical resection of a right proximal humerus sarcoma with brachial plexus neurolysis, reverse total shoulder arthroplasty, lattisimus dorsi, and teres major transfer, myofasciocutaneous advancement to assist with primary wound closure and incisional wound vacuum-assisted closure placement. She tolerated the procedure well and was transferred to the intensive care unit in stable condition. 

**Figure 2:**
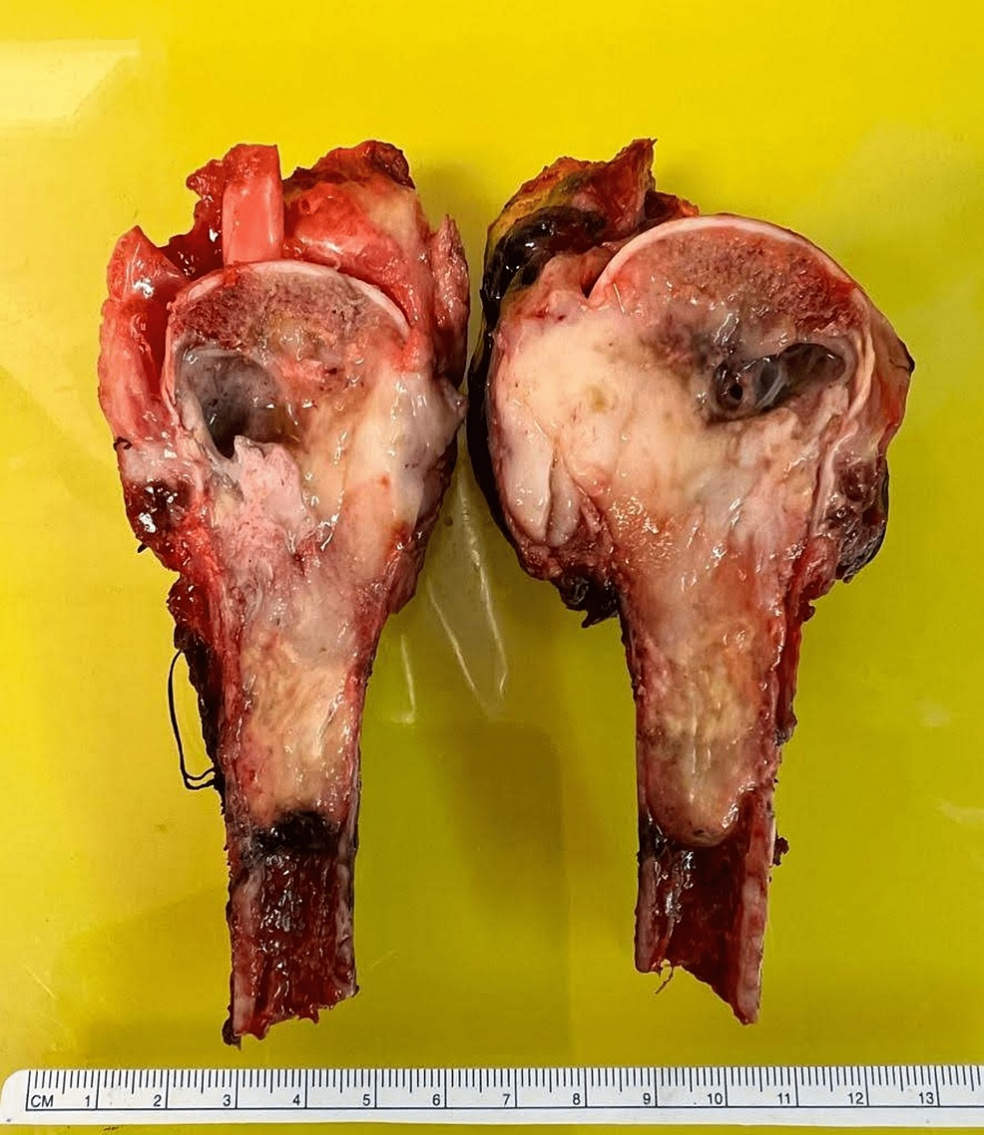
Tumor occupying most of the head and extending distally into a portion of the humerus bone

The humerus head and a segment of humerus bone with attached muscular tissue were sent for pathology examination. The humerus head measured 5.5 x 5 x 4.5 cm, with a piece of humerus bone measuring 6 cm in length. The attached tissue measured 8 x 7 x 5.5 cm. The soft tissue margin was sectioned to reveal a tumor 0.2 cm from the posterior margin and a grossly narrow lateral margin. The humerus head was sectioned perpendicular to reveal a well-defined white tumor measuring 7 x 4.5 x 4 cm, occupying most of the head, and extending distally into a portion of the humerus bone (Figure 2). An area of vascular necrosis surrounded the tumor. The tumor penetrated through the cortex of the head into the posterior/lateral head margin with the involvement of the surrounding tissue. The distal humerus bone was filled with red-brown marrow and had a smooth margin with no gross involvement.

**Figure 3:**
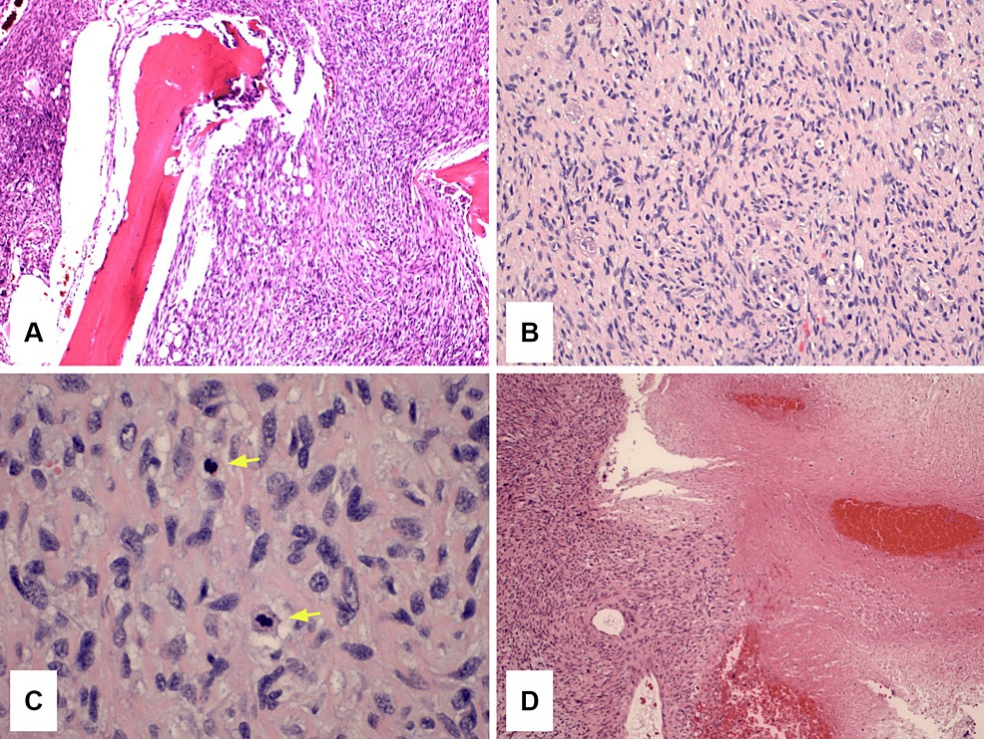
Malignant peripheral nerve sheath tumor of the humerus A) Infiltrating malignant spindle cell tumor with trabecular bone destruction (H&E, 100x); B) Wavy and intersected fascicles of hypercellular and relatively hypocellular areas (H&E, 200x); C) Granular cytoplasm and mitoses (arrow) (H&E, 600x); D) Tumor necrosis illustrated on the right side (H&E, 100x) H&E, hematoxylin and eosin.

**Figure 4:**
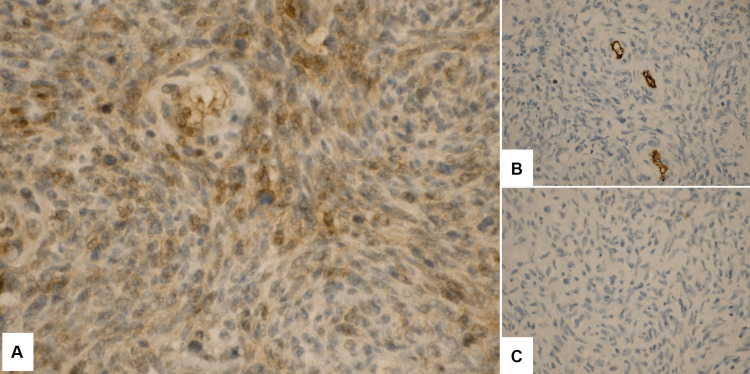
Immunohistochemistry of tumor cells A) SOX10 positive (400x); B) CD34 negative (400x); C) S100 negative (400x)

Histologically, a high-grade sarcoma with a high mitotic rate, microscopic scattered necrosis, and invasion outside of the bone without vascular invasion was identified. The main histologic pattern was suggestive of MPNST, with very cellular to relatively less cellular areas. There was a partial area with less cellularity but more significant nuclear atypia with scattered giant cells and microfocal clear cell pattern (Figure 3). Some of the same IHC stains from the previous biopsy were performed in the current specimen, and tumor cells were seen partly positive for SOX10 while negative for CD34, desmin, S100, and EMA. The final diagnosis by pathology report resulted in an MPNST, classified as T2N0MX, stage IIIA (Figure 4).

The patient was placed in a shoulder immobilizer before being transferred to the intensive care unit. She stated having minimal pain, and sensation to light touch was intact on the dorsal and volar aspect of the hand. The patient was able to move her fingers and wrist actively; however, she had difficulty with thumb extension, grip strength was intact, and the right upper extremity was well-perfused. During her stay, the pain was well controlled; the patient worked with physical therapy. The patient was discharged four days after surgery with hydrocodone, atorvastatin, and aspirin. She could extend her thumb and wrist, make a fist, abduct and adduct fingers, and reported sensation to light touch at median, ulnar and radial nerve distributions to the hand.

The patient was referred to medical oncology and began six weeks of radiation therapy (XRT) a month later. Three weeks after initiating XRT, she had an MRI of the head, which showed no evidence of malignancy. The patient completed XRT, and three months later, she returned to medical oncology; laboratory results included white cell count 3.2 (3.8 - 9.8 K/mm3), absolute neutrophil count 1.9 (2.0 - 7.6 K/mm3), hemoglobin 11.6 (11.2 - 14.9 g/dL), hematocrit 36.5 (33.2 - 43.5 %), platelets 215 (129 - 368 K/mm3), creatinine 0.72 (0.52 - 1.04 mg/dL). An echocardiogram reported left ventricular ejection fraction (LVEF) 55/60% with no abnormal wall motion. The patient started doxorubicin 60 mg/m2, four cycles, every 21 days.

Two months later, the patient was scheduled for a positron emission tomography (PET) scan; she had filgrastim injections at the same time as the PET scan, which revealed a right shoulder prosthesis with uptake in surrounding soft tissue between 3-5, no underlying mass noted. Right pubic bone and right acetabulum standard uptake value (SUV) 6, right upper lobe and right lower lobe nodules up to 0.8 cm with SUV up to 1.8. Laboratories results included white cell count (4.0 K/mm3), absolute neutrophil count (2.5 K/mm3), hemoglobin (11.6 g/dL), hematocrit (36.2 %), platelets (178 K/mm3), creatinine (0.8 mg/dL). Laboratory results can be further visualized in Table 1.

**Table 1:** Laboratory values

	Day 1 of chemotherapy	Two-month follow-up
Leukocyte count (3.8 - 9.8 K/mm3)	3.2	4.0
Absolute neutrophil count (2.0 - 7.6 K/mm3)	1.9	2.5
Hemoglobin, blood (11.2 - 14.9 g/dL)	11.6	11.6
Hematocrit (33.2 - 43.5 %)	36.5	36.2
Platelets (129 - 368 K/mm3)	215	178
Creatinine (0.52 - 1.04 mg/dL)	0.72	0.8

## Discussion

MPNST represents NF's most severe and often fatal complication. This term encompasses previously used designations such as malignant schwannoma, neurofibrosarcoma, and neurogenic sarcoma. These terms imply that the tumors recapitulate or originate from specific nerve sheath cells, such as Schwann cells, perineural fibroblasts, or fibroblasts [4].

MPNSTs comprise approximately 5 percent of all malignant soft tissue tumors and have various origins. Most are derived from neurofibromas or arise de novo in normal peripheral nerves. Schwannoma, ganglioneuroma/ganglioneuroblastoma, or pheochromocytoma origin is infrequent. The majority of MPNSTs show some evidence of Schwann cell differentiation [5]. MPNST may be confused with leiomyosarcoma of soft tissue which is typically a tumor in adults that usually arises from smooth muscle of blood vessels, visceral structures, and the uterine corpus, but can occur anywhere. The growth pattern is primarily fascicular, with the tumor bundles intersecting at wide angles, merging with blood vessel walls. The nuclei are elongated and clefted, and on IHC they show positivity for smooth muscle actin and desmin and are S100 negative, while MPNSTs have wavy, "fishhook" nuclei and fibrillary cytoplasmic processes and are S100 positive [6]. Synovial sarcoma is commonly considered a differential diagnosis of any cellular spindle cell tumor; they usually arise near the knee and ankle joints and around the shoulder and hip. Synovial sarcomas are divided into two groups, biphasic tumors and monophasic tumors. The biphasic tumor comprises an epithelial component with a glandular appearance lined by cuboidal or columnar cells and a sarcomatous component composed of spindle cells with a fibroblast-like appearance. The monophasic synovial sarcoma commonly resembles MPNST as it usually only has the spindle cell sarcomatous component. The immunohistochemical evaluation helps distinguish them; synovial sarcoma is positive for cytokeratin 7, 14, and 19, some cases are positive for EMA, and up to 30% stain for S100 protein. SOX10 appears to be the specific marker of MPNST; synovial sarcomas rarely stain for SOX10 [6,7].

Primary osseous MPNSTs of long bones are rare, with two cases in the femur, two in the ulna, and one in the humerus. They arise from the myelinated intraosseous nerve fibers [8]. The diagnosis of MPNST is based on the following criteria: 1) arises within a peripheral nerve; 2) arises in transition from a benign or other MPNSTs (neurofibroma, schwannoma, ganglioneuroma/ ganglioneuroblastoma, or pheochromocytoma); 3) develops in a patient with NF1 and exhibits the same histologic features as MPNSTs arising from nerves, or 4) develops in a patient without NF1, exhibits histologic features of MPNSTs and shows either or both IHC and ultrastructural features of Schwann or perineurial cell differentiation [5].

MPNSTs typically occur in patients between the ages of 20 and 50 years and are almost equally distributed between the sexes. MPNSTs, both solitary and associated with NF, are rare in children. The mean age of patients diagnosed with these tumors related to NF is lower than that of patients with sporadic MPNSTs. The peak incidence of MPNSTs in NF is during the third decade of life. The peak incidence of sporadic MPNSTs is during the fifth decade of life [4].

Helpful findings for diagnosis include an origin from the nerve, intraneural (intrafascicular) spread within or beyond the primary tumor mass, ganglion involvement, and identification of an associated solitary or plexiform neurofibroma. The majority of MPNSTs are composed primarily of spindle cells. Infrequently, bundles of such cells may show striking variations in cell density, imparting a "tapestry" appearance. The elongated tumor cells often feature hyperchromatic nuclei, are mitotically active, and possess moderate amounts of faintly eosinophilic cytoplasm. Nuclei generally have rounded or tapered ends but are not blunt. In less cellular areas, they sometimes have a wavy contour [5,9].

On IHC, MPNSTs are focally positive for so-called neural markers such as S100 protein, Leu-7, neurofilaments, and myelin basic protein. Although IHC evidence of neural differentiation is documented in more than 50% of patients, it is typically only focal. Neuron-specific enolase cannot be used alone as evidence of neural differentiation because it is frequently positive in many other lesions. SOX10, which is a neural crest transcription factor is expressed in the majority of MPNSTs, including those which are S100 protein negative. In solid uniform positivity for S100 protein cases, other spindle-cell neoplasms (primarily cellular schwannoma) should be excluded before the diagnosis of MPNST is rendered [4]. This tumor was negative for S100 protein; it showed very acceptable morphologic features for this diagnosis, with intersecting fascicles of monomorphic spindle cells having wavy or buckled nuclei associated with cells showing granular cell change. By IHC, H3K27me3 expression was entirely lost, and SOX10 was positive, supporting findings in this morphologic context.

Surgical resection is the main treatment option for MPNST, aiming to obtain a 1-cm margin of clear tissue in all directions. Postoperative external beam radiation therapy is the most popular adjuvant radiotherapy approach; it is used to enhance local control, preserve tissue and function, and reduce local recurrence incidence. Based on 18 trials [3], chemotherapy was associated with a lower risk of local recurrence, and overall survival improved with doxorubicin combined with ifosfamide; also, the absolute risk reduction for death was 5% with adjuvant doxorubicin alone and 11% for doxorubicin combined with ifosfamide.

## Conclusions

This is a case of an intraosseous MPNST of a long bone with a rare site of origin. They usually occur in the mandible, spine, and maxilla; it has only been reported once in the humerus before. Our patient presented with shoulder pain. Even though radiologic findings are often unspecific, and the humerus is an unusual primary site, MPNST should be considered as a differential for prompt management. MPNSTs are highly aggressive, and the treatment for peripheral nerve tumors is surgical resection with or without adjuvant chemotherapy.
